# Factors predicting doctors’ reporting of performance change in response to multisource feedback

**DOI:** 10.1186/1472-6920-12-52

**Published:** 2012-07-10

**Authors:** Karlijn Overeem, Hub C Wollersheimh, Onyebuchi A Arah, Juliette K Cruijsberg, Richard PTM Grol, Kiki MJMH Lombarts

**Affiliations:** 1 IQ healthcare, Radboud University Nijmegen Medical Centre, Nijmegen, The Netherlands; 2 Department of Epidemiology, School of Public Health, University of California, Los Angeles (UCLA), Los Angeles, CA, USA; 3 Center for Health Policy Research, UCLA, Los Angeles, CA, USA; 4 Department of Quality and Process Innovation, Academic Medical Centre, University of Amsterdam, Amsterdam, The Netherlands

**Keywords:** Performance assessment, Mentoring, Multisource feedback, Physicians, Continuous medical education

## Abstract

**Background:**

Multi-source feedback (MSF) offers doctors feedback on their performance from peers (medical colleagues), coworkers and patients. Researchers increasingly point to the fact that only a small majority of doctors (60–70 percent) benefit from MSF. Building on medical education and social psychology literature, the authors identified several factors that may influence change in response to MSF. Subsequently, they quantitatively studied the factors that advance the use of MSF for practice change.

**Methods:**

This observational study was set in 26 non-academic hospitals in the Netherlands. In total, 458 specialists participated in the MSF program. Besides the collation of questionnaires, the Dutch MSF program is composed of a reflective portfolio and a facilitative interview aimed at increasing the acceptance and use of MSF. All specialists who finished a MSF procedure between May 2008 and September 2010 were invited to complete an evaluation form. The dependent variable was self-reported change. Three categories of independent variables (personal characteristics, experiences with the assessments and mean MSF ratings) were included in the analysis. Multivariate regression analysis techniques were used to identify the relation between the independent variables and specialists’ reported change in actual practice.

**Results:**

In total, 238 medical specialists (response rate 52 percent) returned an evaluation form and participated in the study. A small majority (55 percent) of specialists reported to have changed their professional performance in one or more aspects in response to MSF. Regression analyses revealed that two variables had the most effect on reported change. Perceived quality of mentoring positively influenced reported change (regression coefficient beta = 0.527, p < 0.05) as did negative scores offered by colleagues. (regression coefficient beta = −0.157, p < 0.05). The explained variance of these two variables combined was 34 percent.

**Conclusions:**

Perceived quality of mentoring and MSF ratings from colleagues seem to be the main motivators for the self-reported change in response to MSF by specialists. These insights could leverage in increasing the use of MSF for practice change by investing in the quality of mentors.

## Introduction

The assessment of doctors’ professional performance is an important challenge. Nowadays, multisource feedback (MSF) is a central element of these assessments in several countries. Canada was the first country to introduce MSF questionnaires in revalidation programs for doctors [1]. MSF typically involves the completion of questionnaires by a number of colleagues, coworkers and patients – referred to as ‘raters’-, whose responses are summarised to identify the doctors’ performance strengths or weaknesses. Doctors also complete a self-rating using questions identical to those on the colleague survey so that scores can be compared [2]. Questionnaires used in MSF have now been validated for use across a range of specialties in Canada, Denmark, United Kingdom and the Netherlands [3,4]. Although the validity and reliability of multisource assessments have been examined, little attention has been given to the formative aspects of MSF and its likely value for performance improvement. In general, feedback can be beneficial, neutral, or negative in its impact on future practice. Previous meta-analyses and reviews have established broad agreement on characteristics that are likely to make feedback most effective [5-7]. Feedback should focus on task performance, not on judgments about the recipient's character or personality. In addition, it should be specific and clear, since the interpretation of less specific feedback may frustrate the learner.

The impact of MSF on change in practice (referred to as ‘educational impact’) has been subject of several studies. Hall et al in Canada found that 83 percent of participants ‘contemplated’ changing their behaviour^1^, but other studies reported fewer people intending to change their behaviour [8,9]. In a later study in Canada, Fidler et al demonstrated that 66 percent of doctors initiated a change for at least one aspect of performance and this was related to lower mean ratings [10]. Lockyer et al revealed that surgeons reported few change in practice in response to feedback data. They found four factors affecting the likelihood of change in response to MSF: age, the time spent reviewing feedback, surgical specialty and the extent to which self-ratings exceed ratings by others [11]. Studies by Sargeant et al show that physicians believe that patients are most appropriate to assess their practice, and physicians agree most strongly with patient feedback [8]. Some physicians also highlighted they did not implement a change in response to feedback they disagreed with [2].

The educational potential of MSF has been explored by human resources and psychology researchers as well. A study in 2002 showed for instance that women intend to improve more often in response to feedback compared to men [12]. Consistent with studies in medicine, researchers demonstrated that managers who received lower ratings were more likely than others to improve performance [13,14]. In addition, Brett et al observed that overrating (self-rating exceeds MSF ratings by others) limits the use of MSF for future practice [15]. However, if MSF recipients have a good mentor, discrepancies between self-ratings and ratings from others may catalyse a perceived need for change [16,17]. Miller et al concluded in a recent review that MSF could lead to performance improvement, although individual factors and mentoring seem to influence the response [18]. In a previous study we found that mentors who stimulate reflection could increase doctors’ performance change [19]. What is lacking is quantitative empirical evidence confirming the factors found in different studies. Furthermore, the factors identified in business settings such as gender and the discrepancy between self and external assessment, need an evidence base in MSF in the medical profession. On the basis of previous studies and our past experiences, we had the following hypotheses:

1. Personal factors such as higher age, female gender and non-surgical specialty positively affect change in response to MSF.

2. Positive experiences with the MSF assessments related to mentoring and feasibility of webbased service increase change in response to MSF.

3. Lower MSF ratings or a gap between self-ratings and ratings by others positively affect reported change.

In our study, we aimed to answer the following research question: which factors have an impact on specialists’ reported change in response to MSF?

## Method

### Study context

Twenty-six hospitals participated in the MSF system in the Netherlands. In these 26 hospitals, in total 456 medical specialists completed the MSF procedure between September 2008 and December 2010. Besides the collation of MSF ratings from colleagues, coworkers and patients, the complete performance assessment system additionally consists of a reflective portfolio and a facilitative interview with a mentor to increase the acceptance of the feedback and its use for practice improvement. In the reflective portfolio, specialists collected evidence concerning their performance in the seven CanMEDS roles (medical expert, communicator, collaborator, scholar, professional, manager, health advocate) and provided written self-reflections on their performance. Specialists were requested to submit their reflective portfolio to the mentor 2 weeks in advance of the interview. The portfolio and the MSF report were discussed with the mentor (a colleague from a different specialty based in the same hospital) in a facilitative interview. The role of the mentor was to help specialists interpret the feedback, critically analyse their performance and to enhance the use of MSF to guide future performance. Mentors were offered one day of training which included: explanation of the assessment system, goals of the assessment, basic interview skills (active listening) and role-plays. The MSF-system was launched in 2007 in three hospitals and a pilot study established the feasibility of this system [20]. The MSF process is facilitated electronically by an independent webbased service and is described in detail elsewhere [20]. The study was given expedited approval by the Central Committee on Research Involving Human Subjects (known by its Dutch initials, CCMO), the local institutional review board.

### Study design and participants

This study was set up as an observational evaluation study based on questionnaires. We invited all 456 participating specialists to take part in the study. Specialists varied in background and work experience. Half a year after specialists had finished their MSF procedure including a facilitative interview with the mentor, they were asked to complete a questionnaire measuring the self-reported change they made in practice and their experiences with the mentor and the webbased MSF service. We also asked them for permission to use their MSF ratings (their self-ratings and the ratings from colleagues, coworkers and patients) anonymously for research purposes. One reminder was sent to non-responders after three weeks. The questionnaire consisted of eighteen items on a 5-point Likert scale. Participants had the opportunity to explain their answers in detail at the end of the questionnaire.

### Measures

#### Dependent variable

The dependent variable was self-reported change. We measured self-reported change by asking specialists to rate the item: ‘I have changed my professional performance in one or more aspects in the past six months as a result of MSF’ on a 5 point Likert scale (1 = completely disagree, 5 = completely agree).

#### Independent variables

We measured three groups of independent variables: personal characteristics, experiences with the performance assessments and ratings on the MSF questionnaires.

1. Personal characteristics

a) Gender

b) Specialty

We categorised specialties according to specialty type into: 1) non-surgical specialties (internal medicine and subspecialties, paediatrics, dermatology, oncology, psychiatry, radiology, anaesthesiology, pathology and neurology), and 2) surgical specialties (surgery, orthopaedic surgery, urology, gynaecology, ophthalmology, otolaryngology, thoracic surgery, vascular surgery, brain surgery).

c) Years of work experience as a registered specialist.

2. Experiences with performance assessments based on MSF

d) Perceived quality of mentoring

From a previous interview study we developed a scale to measure the quality of mentors in performance assessments. Included items were: preparation of the interview, the degree of increased self-insight and interviewing skills. Responses were invited on a five-point Likert scale. Cronbach’s alpha of this scale was 0.80, establishing its internal consistency.

e) Feasibility of the webbased MSF service

This scale was developed from a previous evaluation study. The scale included: the feasibility, helpfulness of the staff and satisfaction with the webbased service. The items used five-point Likert scales ranging from 1 (completely disagree) to 5 (completely agree). Analysis confirmed the internal consistency of this scale with a Cronbach’s alpha of 0.72.

3. Feedback ratings on MSF questionnaires

f) Mean MSF ratings from colleagues. We calculated for each specialist a mean score of all colleagues’ ratings on the MSF questionnaires.

g) Mean MSF ratings from coworkers. We calculated for each specialist a mean score of all coworkers’ ratings on the MSF questionnaires.

h) Mean MSF ratings from patients. We calculated for each specialist a mean score of all patients’ ratings on the MSF questionnaires.

i) Self-ratings. We calculated for each specialist a mean score of all self-rated items on the MSF questionnaires.

j) Discrepancy between self-rating and ratings by others. We calculated a mean gap score by subtracting mean ratings between all raters per specialist from the self-rating by specialists.

### Statistical analysis

Descriptive statistics were calculated for the three categories of independent variables. Sum-scores were calculated for the two subscales on perceived quality of mentoring and feasibility of the webbased service. MSF ratings by colleagues, coworkers and patients from male and female specialists were compared using unpaired t-tests and one-way ANOVA. A p-value < 0.05 was considered significant.

After the initial analyses the independent variables were tested for univariate relationships with self-reported change in order to select the items for the multivariate analysis. The relationship between self-reported change (a score on a 5-point Likert scale and thus considered as a continuous variable) and the dichotomous variables (gender and speciality) was analysed with the Mann–Whitney U test. The correlation between the other variables and self-reported change was analysed with Pearson’s correlation. Variables with p ≤ 0.15 were found to be eligible for multivariate regression analysis. Multiple regression analysis was used to examine which of the independent variables are decisive in doctors’ reported change. The specialists being anonymous, we could not correct for the nesting of specialists within hospitals and specialist groups with a multi-level analysis. We selected backward regression as the multiple regression method. The criteria for entry and removal were .05 and .10 respectively, with listwise exclusion of cases. We used SPSS, version 18.0.1 for the statistical analysis.

## Results

### Study participants

A total of 236 specialists responded to the survey of a possible 452 (52 percent). Seventeen of the non-responders indicated they had lack of time to complete the questionnaire. Because of anonymity issues, other reasons for non-response could not be retrieved. The participants consisted of 144 men (61 percent) and 92 women (39 percent). The percentage of female specialists reflects the whole population of specialists in the Netherlands well [21]. Specialists participating had on average 14 years of work experience.

### MSF ratings and self-reported change

The mean gap between the self-ratings and the colleagues’ ratings was −0.21 with a range from −2.34 to 1.81, which means that on average specialists rate themselves less positive than their colleagues rate them. However, 30.3 percent of specialists were over-raters. Female specialists scored themselves significantly lower compared to male specialists on the self-assessment (t = −3.2, p < 0.05). However, scores from colleagues, coworkers and patients revealed no significant differences based on gender of the specialist. Analysis of variances of the mean gap between colleagues’ ratings and self-ratings’ revealed that female specialists were significantly more often under-raters (F = 3.986, p < 0.05.) compared to male specialists. A small majority (55 percent) of doctors involved believed that they succeeded in improving their performance as a result of the MSF assessments.

### Self-reported change and relationship with the independent variables

Univariate analysis using Mann Whitney U tests for the first group of independent variables, gender, specialty and years of work experience, revealed that none of the personal variables, were significantly associated with self-reported change (see Table 1 for p-values of the correlations).

**Table 1:** Personal characteristics and correlation with reported change

**Domain**	**Percentage**	**Mean**	**SD**	**p**
- male (n = 144)	61 %	-	-	0.459
-female (n = 92)	39 %			
1. NON-SURGICAL SPECIALTY: dermatology (n = 8), cardiology (n = 4), pulmonology (n = 5), internal medicine (n = 40), psychiatry (n = 5), neurology (n = 13), paediatrics (n = 26 ), anaesthesiology (n = 19), radiology (n = 17) and all laboratory specialties such as medical microbiology, pathology and clinical chemistry (n = 46).	71 %	-	-	0.403
2. SURGICAL SPECIALTY: general surgery (n = 17), urology (n = 5), orthopaedics (n = 6), gynaecology (n = 15), ophthalmology (n = 2), otolaryngology (n = 8)	29 %			
Years of work experience	-	14.4	8.18	

In the second category, both perceived quality of mentoring (r = 0.565, p <0.01) and feasibility of the webbased service (r = 0.169, p <0.01) were positively associated with reported change and therefore eligible for multivariate analysis. (See Table 2 for p-values and correlations).

**Table 2:** Experiences with performance assessments based on MSF and correlation with reported change

**Variable**	**Mean**	**SD**	**Pearsons’ Correlation Coefficient**	**p**
Mentor supervision quality scale	3.49	0.85	0.565	0.000*
Feasibility of webbased service	3.09	0.97	0.169	0.006*

*Variables with P ≤ 0.15 were included in multivariate analysis.

Among the last category of variables including MSF ratings, only the mean ratings of colleagues (r = −0.195, p <0.01) and the mean of self-ratings (r = −0.179, p <0.01) were significantly correlated with reported change and therefore eligible for multivariate analyses.

Both these latter correlations were negative, which means that higher self ratings (i.e. more positive) and higher MSF ratings by colleagues (i.e. more positive) were associated with less self-reported change. (See Table 3 for correlations and p-values)

**Table 3:** Scores on MSF and correlation with reported change

**Variable**	**Mean**	**SD**	**Pearsons’** **Correlation Coefficient**	**p**
Mean ratings from colleagues	8.37	0.69	−0.195	0.005*
Mean ratings from coworkers	8.33	0.48	0.020	0.413
Mean ratings from patients	8.12	0.44	−0.013	0.438
Mean of all self-rated items	8.11	0.49	−0.179	0.009*
Discrepancy between self-rating and ratings by colleagues	−0.21	0.62	−0.023	0.384

*Variables with P ≤ 0.15 were included in multivariate analysis.

After testing for univariate analysis, only the above mentioned four out of the in total nine variables were selected for multivariate analysis. Within the corresponding multivariate analyses with backward selection, two of the four variables were found to predict self-reported change. First, perceived quality of mentoring (standardized regression coefficient beta: 0.552, p < 0.05) seems to positively influence the change reported by specialists. Second, the mean MSF ratings by colleagues (standardized regression coefficient beta:-0.152, p < 0.05) affects reported change directly. The explained variance of these two factors combined was 34 percent. This implies that higher mean MSF ratings (i.e. more positive) by colleagues, made it less likely that a specialist will report change after the MSF assessment. (See table 4 for the results of the regression analyses).

**Table 4:** Results of multiple regression analysis of four independent variables and reported change

**Independent variables**	**Standardized Beta**	**T**	**p**
Feasibility webbased service	−0.102	−1.557	0.121
Mean ratings from colleagues	−0.157	−2.414	0.017*
Mean of al self-rated items	0.055	0.851	0.396
Mentor quality	0.527	7.960	0.000*

* = p < 0.05.

## Discussion

### Main findings

Our national survey succeeded in obtaining specialists’ views on change in practice as a result of MSF assessments and investigating the association between self-reported change and different independent variables. With regard to the key ingredients that determine practice improvement in response to MSF, perceived quality of mentoring was the main motivator in this study with a limited number of independent variables. Furthermore, this study shows that specialists who receive lower ratings from their peers (medical colleagues) tend to report more change in practice. As a finding of serendipity, we found that female participants are significantly more often under-raters and score themselves lower compared to their male colleagues.

### Comparison with other literature

The importance of mentoring for doctors’ performance change in response to MSF is supported by earlier work in this domain. In our previous qualitative study on hospital-based assessments we showed that the use of MSF depends on a combination of concrete goals, mentoring and structured follow up [19] Based on recent literature, we expected other variables such as gender, specialty, work experience and the discrepancy between self-ratings and ratings by others to be influencing factors as well. This was not confirmed by our current study. Presumably, differences in change with MSF amongst Dutch medical specialists are not based on gender or work experience.

The fact that specialists who receive lower MSF ratings from their colleagues, tend to improve more is in line with earlier studies in business settings as well as in medicine [11,14]. However, only a small majority of specialists (55 percent) reported to have changed. In two previous studies, 66 percent of doctors intended to change or reported to having initiated a change [11,20]. There are several possible explanations which may account for the fact that less specialists reported change. First, the MSF reports offered to specialists contain means and standard deviations which were in a relatively narrow range and therefore it was difficult for specialists to identify areas for improvement. This might be caused by the fact that Dutch specialists receive less critical feedback compared to other countries. Second, a comparison with their peer group was not provided in their MSF reports and therefore some specialists may not have considered their ratings as a need for improvement. Third, Dutch doctors might experience less urgency to change compared to doctors in other countries. Our study revealed that MSF ratings by coworkers and patients are not decisive in specialists’ change in response to MSF. This is in contrast with a study by Fidler et al in 1999. They showed that those physicians who reported to change received significantly lower mean ratings (i.e. more negative) from patients and also from peers, referring physicians and coworkers [11]. In a previous study, we found that specialists are more satisfied with MSF containing narrative comments [20]. In this study, we found that MSF ratings from colleagues are a predictor of specialists’ reporting of a change in performance. A possible explanation could be that colleagues provide more often narrative comments to explain their ratings compared to coworkers and patients. However, this hypothesis deserves further study. Finally, the finding that men are more often over-raters compared to their female colleagues is in agreement with earlier findings in human resource studies. Atwater et al found that men tend to overrate themselves more often compared to woman [22].

### Strengths and weaknesses

We consider the findings of this study in the light of potential study strengths and limitations. This study adds to the literature on MSF by moving beyond qualitative research to an empirical analysis of the influence of various factors on doctors’ reported change. Strengths of this study are the anonymity of the questionnaire, reducing the likelihood of socially desirable answers as well as the large sample size. The questionnaire being anonymous, specialists’ age and more important the hospital and specialist group they are based in were not available for analysis. Caution is therefore required in interpreting the findings of our study as it was not possible to include other important predictors such as culture of the specialist group. It was therefore also not possible to correct for nesting of specialists within specialist groups with a multilevel analysis. Future research is necessary to investigate other predictors of change in response to MSF in more detail including the effect of culture in various specialty groups on reported change as these factors have been found to be important determinants in previous studies [18,19], What also would have been an important variable to compare is the effect of various combinations of specialists and mentor based on gender and specialty, as this match probably plays a role in effective mentoring [23]. Furthermore, because of the anonymity of the data from specialists participating in the project, we were not able to compare responders with non-responders to see if the group of responders was a representative sample with respect to mean ratings. In addition, an important limitation of this study concerns the response rate of 52 percent. Returning a questionnaire reflects in itself a certain level of ‘openness to change’ which might have contributed to the positive nature of our findings. Finally, we measured specialists’ self-perceptions of their reported change and we did not check with external assessments or observations by others whether these changes had taken place.

### Importance for future research and practice

Our findings have several implications. The main finding of this study is that the perceived quality of mentoring is the most significant predictor of doctors’ reported change. In contrast, it is well known that many doctors do not actively seek a mentor of their own accord and women have more difficulty in finding a mentor than their male colleagues [24]. In light of the increased prominence of underrating in women, this is even more disappointing. Mentoring should not be left entirely at the initiative of doctors but it should be a structural part of MSF programs. In a previous study, we investigated which strategies mentors use to achieve that doctors integrate external feedback in their self-concepts [25]. These strategies were: collating and contrasting information, posing reflective questions and goal setting. An important implication of this study is that mentors should be well-equipped to perform this role and therefore additional training with an emphasis on these strategies might be useful.

Further avenues for future research are clearly signposted from this study. First, studies investigating real change in practice in response to MSF, for example as observed by others instead of self-reporting by specialists, are necessary to verify our findings. For example longitudinal MSF scores can be compared. Furthermore, a more detailed understanding of the mentor-mentee relationship and its effect on self-assessment would also be valuable. For instance, it is not yet clear how often facilitative interviews with a mentor should occur for an optimal effect. Additionally, our findings warrant other studies to determine how MSF data can better highlight the need to improve. Presumably, narrative comments play a role in this and this should be further investigated. We join Archer and Miller [10] in advocating for studies over extended periods in which matched groups of doctors are opposed to different interventions.

## Conclusions

This study demonstrates that the perceived quality of mentoring and MSF ratings from colleagues are important motivators for specialists’ performance change in response to MSF. As we could only include a limited number of variables, other motivators might also have an effect on doctors’ change in performance in response to MSF. Socio-demographic variables such as gender and age do not influence the use of MSF for further change. Hopefully, the results of our study will encourage other countries to establish structural mentoring in MSF as doctors will not actively seek a mentor of their own accord to discuss MSF with and use the MSF for change in practice.

## Competing interests

The authors declare that they have no competing interests.

## Authors’ contributions

KO was the primary investigator of the study. KO, KML and HCW conceived and designed the experiments. KO, KML, JC and OAA analyzed the data. KO, JC and OAA contributed analysis KO drafted the article and KML HCW RPTMG OAA and JC revised the article critically for important intellectual content and approved the final version to be published.

## Pre-publication history

The pre-publication history for this paper can be accessed here:

http://www.biomedcentral.com/1472-6920/12/52/prepub
